# The Correlation between Dietary Selenium Intake and Type 2 Diabetes: A Cross-Sectional Population-Based Study on North Chinese Adults

**DOI:** 10.1155/2020/8058463

**Published:** 2020-01-16

**Authors:** Sultan Mehmood Siddiqi, Changhao Sun, Xiaoyan Wu, Imranullah Shah, Anam Mehmood

**Affiliations:** ^1^Department of Nutrition and Food Hygiene, College of Public Health, Harbin Medical University, Harbin, Heilongjiang Province 150081, China; ^2^Department of Clinical Psychology, College of Public Health, Harbin Medical University, Harbin, Heilongjiang Province 150081, China

## Abstract

The relationship between selenium (Se) and type 2 diabetes (T2D) remains controversial. In previous animal and cell studies, Se was found to be insulin mimic and antidiabetic, whereas recent epidemiological and interventional trials have shown an unexpected association between high Se intake and increased risk of T2D. The present study aimed to investigate the significance of dietary Se and T2D in North Chinese adults. A large sample of the population was enrolled through cluster sampling in Northern China (*N*=8824). Information on basic characteristics, anthropometric measures, and dietary Se intake was collected from each subject for analysis. Multivariable logistic regression was used to investigate the association between dietary Se and T2D through adjusted odds ratio (OR) and the corresponding 95% confidence interval (CI). The average nutritional Se intake was 52.43 *μ*g/day, and the prevalence of T2D was 20.4% in the studied population. The OR for developing T2D was 1.66 (95% CI: 1.38, 1.99; *P* for linear trend <0.005), comparing the highest to the lowest quintile of energy-adjusted Se intake in multivariate logistic regression analysis. The mediation analysis discovered that glucose metabolism (indicated by FBG and HbA1c) mediated this association. In conclusion, our research adds further support to the role of high dietary Se in the incidence of T2D. The results also suggested that this association was mediated by glucose metabolism.

## 1. Introduction

Se is an essential micronutrient that plays structural and enzymatic roles in antioxidant defense systems, such as metabolism of thyroid hormone, red-ox homeostasis, reproduction, and immunity through Se-containing selenoproteins such as iodothyronine deiodinases, glutathione peroxidases (GPx), and thioredoxin reductases [1–3]. It has the potential to prevent the onset and progress of T2D, gestational disease, cardiovascular disease (CVD), [4] colorectal cancer, and prostate cancer by the action of its antioxidant selenoproteins and insulin mimic selenate [4–7]. Various prospective studies have claimed the reduction of diabetic risk with higher Se intake, suggesting a beneficial effect of Se supplementation [7, 8]. Likewise, individuals with higher Se levels have a lower chance of T2D, as claimed in a prospective study on healthy men and women in the US population [7]. Furthermore, the higher intake of inorganic Se has a progressively negative association with T2D in various animal studies [9, 10].

However, some studies reported a nonsignificant association between Se and T2D [11, 12]. A cross-sectional survey on the Nord-Trondelag Health Survey found no evidence for the significant association of serum Se and T2D by analyzing the trace elements status in the blood of diabetic patients [13]. In contrast, other reports have significantly proved a positive association between Se and T2D [14, 15]. The National Health and Nutrition Examination Survey of US (NHANES) reported an increased risk of T2D with the increase of serum Se in 1988–1994 [16] and further in 2003-2004 [14]. Our findings are also supported by a large sample ORDET cohort study of Northern Italian women that confirmed a significantly positive correlation between serum Se and T2D [17]. Likewise, a cross-sectional survey on the Asian population further confirmed that high dietary Se might be associated with the possible risk of T2D [18]. Besides, rats, mice, and pigs reported diabetic phenotypes with high Se diet (0.4 to 3.0 mg/kg) [19]. Moreover, the findings of an observational study on a large sample of Chinese adults reported that Se supplementation might be positively connected with T2D [20].

Natural foods are enriched sources of Se such as cereals, seafood, organ meat, and crops. In China, Se intake tremendously varies among populations. For instance, Se is deficient in Europe but very high in Canada, Venezuela, the US, and Japan. Se level in the soil is affected by pH, pesticides, rain, and evaporation. Dietary intake of Se considerably varies in the world due to inconsistent Se contents of plants, animal foods, and soil [21]. Se contents in a particular food depend upon the Se level in the soil where the food is grown, and the average recommended Se intake depends upon glutathione peroxidase potential activity [22]. Se intake in the United States is 60–220 *μ*g/day, and the health benefits or metabolic toxicity of Se are not very clear at higher Se intake, more than the recommended dietary allowance (RDA) [21, 23]. In Europe, Se intake is comparatively lower than in the US but varies considerably between marginally adequate to adequate (30–90 *μ*g/day) in Western and Central Europe and deficient to inadequate intake (7–30 *μ*g/day) in Eastern European regions [21]. Few Chinese people studies have adjusted necessary dietary information and studied the association between dietary Se and T2D. Therefore, this study investigated the possible correlation between dietary Se and T2D in North Chinese adults.

## 2. Materials and Methods

### 2.1. Study Population

The Harbin Cohort Study on Diet, Nutrition, and Chronic Noncommunicable Diseases (HDNNCDS) is a population-based study launched at the nutrition and food hygiene department of Harbin Medical University by using similar survey methods as described in previous publications [24]. In this cross-sectional study, well-trained personals interviewed all the participants and collected demographic and health-related data by using a well-structured questionnaire during a medical examination. On that account, a total of 42 communities were indiscriminately selected from 3 strata in 7 urban administrative regions of Harbin, representing the baseline status of geographical atmosphere, economic situation, and social behavior. We decided the eligible participants (1) between 20 and 74 years, (2) residing in Harbin for not less than two years, and (3) without type 1 diabetes or cancer. Finally, 9,828 people participated in this study among 12,865 eligible participants, with a 76.4% participation rate. Reasons for not joining were unconcerned in the survey (4.6%), restraints of time (7.8%), poor study understanding (10.1%), and no reason is given (1.1%). 94 individuals were further excluded at baseline interview, who were found to be more or less than 74 and 20 years, respectively, and also those who had reported for extreme consumption of energy (men >4,200 or <800 kcal/day, women >3,500 or <500 kcal/day, *N*=724) and missing data for body mass index (BMI), education, waist circumference (WC), and Se intake (*N*=186). After all these exclusions, the final study comprised of 8,824 subjects (3,187 men and 5,637 women). This study had been registered at chictr.org as ChiCTR-ECH-12002721.

### 2.2. Measurements

Relevant survey information was obtained in this cross-sectional study through structured questionnaires including (1) dietary habits, (2) physical examination and lifestyle, (3) and demographic measurements. A semiquantitative food frequency questionnaire model was used to evaluate the average daily dietary consumption rate over the past 12 months. This model included 14 different food groups, with 103 most popular and common food items consumed by Harbin people. We used Food Nutrition Calculator (V1.60, Chinese Center for Disease Control (CDC), Beijing, China) to estimate dietary Se intake (*μ*g/day) and total energy consumption (kcal/day) in different food items [25].

Well-structured lifestyle questionnaires were established to collect thorough information on disease history, family history of T2D (yes/no), age, education level (nonformal education, education in elementary school, middle or high school education, education in technical school or college, and university education), exercise (any kind of sports other than walking), cigarette smoking (smoked not less than 100 cigarettes over lifetime), alcohol consumption (consumed at least a bottle during last year), Se deficiency and supplementation history, occurrence of hyperlipidemia, coronary heart disease (CHD), and hypertension (systolic blood pressure (SBP) ≥ 140 mmHg or diastolic blood pressure (DBP) ≥ 90 mmHg, and/or taking medications for hypertension).

Anthropometric measurements, including body weight, height, waist circumference (WC), and blood pressure (BP), were examined by well-trained personals. The average of three measurements was calculated with respective accuracy of 0.1 kg, 0.1 cm, 0.1 cm, and 1 mmHg by using standard conventional procedures. Body mass index (kg/m^2^) was measured by dividing the weight (kg) with a square of the height (m^2^). The fat mass (FM) analyzer analyzed the body fat by using the electric impedance method (OMRON HBF-306, Omron Corporation, Dalian, China).

Homeostasis model assessment of insulin resistance (HOMA-IR) was calculated with the formula: fasting glucose (mmol/L) × fasting insulin (mIU/L)/22.5, and HOMA-beta was calculated according to the method: 20 × fasting insulin (mIU/L)/FPG (mmol/L)−3.5 [26]. Moreover, the haemoglobin A1c (HbA1c) assays were carried through using high-performance liquid chromatography (HPLC) on a Bio-Rad Variant VCS Hemoglobin Testing System (Shiga, Japan). The HbA1c intra-assay and interassay coefficients of variation were 0.7% at a value of 8.0% and 1.2% at a value of 5.8%.

An OGTT was performed as per World Health Organization guidelines (WHO) [27]. Fasting and postprandial blood samples (blood samples taken after drinking water containing 75 grams of glucose) were collected with and kept at room temperature for 30 minutes. Samples were centrifuged for 5–10 minutes at a speed of 1,000 rpm/min, and separated serum was collected for analysis. Incidence and prevalence of T2D were examined as follows: (1) by asking questions like “When the diagnosis confirmed the disease?” and “When were you diagnosed?” and “Did you control diabetes?” or (2) fasting blood glucose (FBG) ≥ 7.0 mmol/L, and/or (3) 2 hr glucose ≥ 11.1 mmol/L or (4) using diabetic medications.

### 2.3. Statistical Analysis

Quantitative and qualitative data were expressed in terms of mean ± standard deviation (SD) and percentage, respectively. The study population was classified into energy-adjusted quintiles of dietary Se intake by using the residual method: ≤39.24, 39.25–46.45, 46.46–52.57, 52.58–60.68, and ≥60.69 *μ*g/day. One-way variance analysis (uniformed data) and Kruskal–Wallis H-test (abnormal data) evaluated the divergences in the continuous data. Differences in qualitative data were measured by using the *χ*^2^ test. Logistic regression analysis assessed the ORs or the relative risk for developing T2D with 95% CI by comparing the quintiles of energy-adjusted Se with the lowest quintile serving as a reference. We used two core adjustment models: model 1 (partially adjusted model) adjusted for age, sex, BMI, waist; model 2 (fully customized model) additionally adjusted for smoking (past, current, never), alcohol intake, energy intake (nonalcohol), body fat, education, family history of diabetes, exercise, hypertension, and coronary heart disease. Logical regression analyzed the linear trend with Se as a median variable of each category. Subgroup analyses investigated the possible interaction between energy-adjusted Se and gender or BMI. Fully adjusted ORs with potential 95% CIs were evaluated in the sex (male and female) and BMI subgroup (<25 or ≥25).

The variations of insulin resistance in different intakes of dietary Se were analyzed across quintile of dietary Se. HOMA-IR and HOMA-*β* were compared among groups using ANCOVA after controlling for age, sex, and BMI. Partial correlation between energy-adjusted dietary Se and markers of glucose metabolism (FINS, FBG, and HbA1c) was compared using ANCOVA after controlling for age, sex, and BMI.

Mediation analysis was performed to evaluate the role of HOMA-IR, HOMA-*β*, FBG, FINS (fasting insulin), and HbA1c as potential mediators of the association between dietary Se and T2D. And statistical significance for the mediation effect was carried out by formally testing for the average causal mediation effect, using bootstrapping techniques. The complete data analyses were carried out through SPSS 17.0 and R version 3.0.3 (http://www.r-project.org/), while *P* < 0.05 was regarded to have statistical significance.

## 3. Results

The mean dietary Se intake of 8,824 participants was 52.43 *μ*g/day, indicating a relatively high baseline Se level. We observed that the participants with T2D were older and most likely the male with a higher level of BMI, body fat, protein, TG, cholesterol, energy intake, CHD, hypertension, obesity, and lower levels of carbohydrate than those in healthy individuals while nonsignificant differences were observed for fiber, cholesterol, and drinking (Table 1).

Wheat, livestock, fish, rice, and eggs are primary sources of dietary Se intake in the studied population (Table 2). Significant differences were assessed across quintiles of energy-adjusted Se intake in terms of BMI, sex, education, exercise, WC, consumption of alcohol, smoking status, and body fat. Meanwhile, age, energy intake, family history of diabetes, coronary heart disease, hypertension, hyperlipidemia, HOMA-IR, and HOMA-*β* showed a nonsignificant association with energy-adjusted Se groups (Table 3).

The age, sex, BMI, waist adjusted OR for the highest quintile was 1.74 (95% CI 1.46–2.07) when compared with the lowest quintile of energy-adjusted Se. It reveals a progressive increase in the risk of T2D (*P*_trend_ < 0.001). After multivariate adjustment for age, sex, BMI, waist, smoking (current, past, never), alcohol consumption, total energy intake, level of education, body fat, exercise, hypertension, family history of diabetes, and CHD, the adjusted OR for Q2, Q3, Q4, and Q5 were 1.34 (95% CI 1.11–1.63), 1.20 (95% CI 0.99–1.47), 1.39 (95% CI 1.14–1.69), and 1.66 (95% CI 1.38–1.99), respectively (Table 4). Seemingly, the adjusted OR is greater than 1, indicating a positive correlation between dietary Se intakes and T2D. While exercise is significantly associated with dietary selenium, it does not affect the association between dietary selenium and T2D. Table 4 explains the outcomes of subgroup analysis. It was observed that dietary Se intake was positively correlated with T2D in gender and BMI subgroups (*P*_trend_ < 0.05), indicating a risk factor for both genders.

The correlations between energy-adjusted dietary Se (*μ*g/day) quintiles and HOMA-IR and HOMA-*β* are presented in Table 5. Both HOMA-IR and HOMA-*β* were nonsignificantly (*P* > 0.05) correlated with energy-adjusted Se quintiles after adjusting for age, sex, and BMI.

Table 6 explained the relationship of energy-adjusted dietary Se with HbA1c, FBG, and FINS (fasting insulin). It was observed that energy-adjusted dietary Se was directly correlated with HbA1c and FBG (*P* < 0.001). On the other hand, we did not find any significance for the level of FINS (*P* > 0.05).

In mediation assessment, we observed statistically significant mediation effects of FBG and HbA1c (0.5498095% bias-corrected intervals: 0.29877, 0.86451, *P* ≤ 0.001 for FBG, and 0.79426, 95% bias-corrected intervals: 0.41340, 2.41389, *P*=0.006 for HbA1c), suggesting that the association between dietary Se and T2D was potentially mediated by glucose metabolism while no statistically significant mediation effect was found for FINS, HOMA-IR, and HOMA-*β* (Table 7).

## 4. Discussion

We observed a significant linear association between dietary Se intake and an increased risk of T2D. This observational study was carried out in a large sample of the population who belongs to Harbin city in Heilongjiang province of Northern China, comprising 8,824 adults between the ages of 20 and 74 years. Several studies found that healthy people have higher Se levels than people with T2D [6, 7, 28, 29]. Various interventional and epidemiological studies also drew conflicting conclusions on the association between T2D and Se level in different biomarkers [7, 23, 30, 31]. In a double-blinded, randomized, placebo-controlled model, the subjected population was supplemented with 200 *μ*g of Se intake for consecutive six weeks starting from 24–28 weeks of gestation. Consequently, a protective effect was demonstrated on levels of high sensitivity C-reactive protein, glucose metabolism, and oxidative stress biomarkers [5]. The finding of Suresh et al. [32] further explained these observations by indicating that decreased level of serum Se may lead to tissue destruction and inflammation in patients with T2D. An animal trial investigated that high Se exposure may induce reverse regulation of reactive oxygen species, demonstrating a hepatic insulin resistance [33].

In contrast, recent findings from cross-sectional studies in China [18], prospective studies in Europe [17], and observational and clinical outcomes from the US [16] indicate that Se supplementation or high Se level might be the possible risk of T2D. Various studies observed the association between Se intake (supplementary or dietary) and T2D with inconsistent conclusions. The previous three cross-sectional studies concluded mixed results [14, 34, 35]. Only one study supported our findings and investigated the possible risk of high dietary Se in the prevalence of T2D in Chinese people [35]. Moreover, a randomized, placebo-controlled study explores the adverse impact of Se supplementation on glucose homeostasis in diabetic patients [36]. The third National Health and Nutrition Examination Survey (NHANES III) [16] and NHANES 2003-2004 [14] estimated a risk factor of T2D with high Se level. A previous meta-analysis [37] further confirmed this association, although it fails to reach statistical significance, with pooled relative risk 1.06 (95% CI 0.97–1.15). Contrarily, several studies assessed a nonsignificant association between Se and T2D [38, 39]. A PRECISE pilot trial in the UK evaluated that intervention with 100, 200, or 300 *μ*g/day of Se for six months did not show any diabetic effects in participants with relatively low Se levels [39]. Nevertheless, the outcomes of Se and Vitamin E in Cancer Trial also (SELECT) confirmed a nonsignificant association between Se supplementation and T2D [16].

Various cross-sectional studies [14, 40, 41], longitudinal analyses, and controlled trials [35, 42, 43] support the findings in the present study, suggesting a statistically significant positive correlation between dietary Se and T2D. Moreover, a cross-sectional survey on Asian population further explored a decisive significance for the prevalence of T2D with high dietary Se intake [18].

The dietary Se is nonsignificantly associated with HOMA-*β* and HOMA-IR in the present study. The association between Se nutritional status and insulin resistance is very complicated and intriguing. A negative association was found between Se and HOMA-IR in Korean [44] and Turkish populations [45]. In contrast, serum Se and HOMA-IR reported a positive correlation in obese Egyptian children [46] and elderly Polish men with MS [47]. However, a nonsignificant association was found between serum Se and insulin resistance in a Swedish study [48]. The present study revealed that the relationship between dietary Se and the prevalence of T2D might be mediated by glucose metabolism instead of insulin resistance.

There is limited mechanistic evidence that may clarify the relationship between increased Se exposure and high risk of T2D; therefore, at present, any such discussion is highly speculative. Se has high interindividual variability and narrow therapeutic range in terms of metabolic sensitivity [49, 50]. Se forms such as selenate and selenite may impair the insulin signaling in rats and stimulate the catabolic activity in muscles with the increase of glycogen consumption and glycolysis rate [51]. High dietary Se may stimulate overexpression of glutathione peroxidase-1 (GPx-1) and other antioxidant selenoproteins, promoting insulin resistance and obesity [52–54] or may induce the release of glucagon resulting in hyperglycemia [55]. Likewise, in humans, a group of nondiabetic pregnant women showed a strong positive association between GPx activity and insulin resistance [43]. From a mechanistic explanation, Se intakes higher than the recommended level for optimal antioxidant activity of selenoproteins, such as glutathione peroxidases (55 *μ*g/day, serum or plasma concentrations 70–90 *μ*g/L) [22, 56], will lead to the nonspecific binding of selenomethionine substituting methionine in albumin and other proteins [3]. Other Se-dependent proteins, including selenoprotein P [57, 58], methionine-R-sulfoxide reductase B1 [59], thioredoxin reductase 3 [59], and selenoprotein S [60], may also be involved in glucose metabolism or T2D. The metabolic pathways involved in this extra Se pool have not been fully understood and may be responsible for some of the adverse effects of high Se exposure on glucose metabolism.

In this study, we observed that Se has a significant positive association with the level of HbA1c and FBG. On the other hand, we did not find any changes at the level of FINS. Our results are supported by a recent population-based study [61], suggesting that subjects in higher quintiles of serum Se had an increased risk of T2D (higher levels of FBG and HbA1c) compared with those in the first quartile. However, FBG reflects the glycemic status and HbA1c is regarded as a more reliable marker since it indicates long-term changes in the glycemic control. Thus, the impact of Se on HbA1c levels observed in this study confirms that Se is involved in the metabolism of glucose. Another study investigated the association between high serum Se levels and impaired fasting glucose and elevated fasting serum glucose [20]. Several studies reported that patients with T2D had higher serum Se concentration compared with healthy subjects, and serum Se levels had positively correlated with plasma glucose in T2D [14, 16, 42]. The link between Se and glucose seems to be very complicated and is supposed to be reflected by a nonlinear U-shaped dose-response relationship [62].

Moreover, a comprehensive study of the relationship between Se and T2D [63] concluded that this complicated relationship could be interpreted as a potential hazard of optimal activity of some or all Se-containing proteins at different physiological ranges. Current and previous studies have been conducted in participants with an average dietary Se intake close to the recommended nutritional intake, but this association may differ among people with deficient nutritional Se intake. Results of previous investigations intimated a U-shaped correlation between dietary Se and T2D. Thus, an imbalance in the physiological range of dietary Se might be a risk factor for the prevalence of T2D. A recent study on US adults explored the risk of supplementary Se, but this statistical significance was only confirmed in males in NHANES III [14].

The main strength of this survey is that it is the first and most extensive study to date to study the correlation between dietary Se intake and T2D of more than 8,800 people in Northern China. Most of the previous studies confirm this association in Europe or the US. Notably, it is utile to conduct this study on the north Chinese population because results may be affected due to differences in culture, geography, and dietary habits. Most strikingly, it is the first cross-sectional design to inquire about the link between dietary Se intake and T2D with large-scale north Chinese population and consistently supported by previous outcomes.

However, this study also has some limitations. First, a causality of relationship is not well defined in this cross-sectional evaluation, so there is a need to verify our outcomes through additional prospective designs. Second, it was hard to measure the Se level in plasma or serum due to limited resources. However, in the environment with substantial changes in Se content of food and high frequency of using mineral and vitamin supplements, the use of questionnaire data to estimate Se intake has attracted attention. First, HDNNCDS developed a detailed database to analyze the Se content of local foods with *ad hoc* measurements. Secondly, the range of dietary Se intake in this study is consistent with the previous measurement results of Se status of the Chinese population-based on biomarkers [64]. It is easier to estimate Se intake through questionnaire survey data, while previous studies have analyzed the Se contents and Se status in various foods from all over China with minimal variability [18, 65]. Finally, [24] no user of Se supplements was detected in our survey. In our settings, the evaluation of Se exposure by assessing dietary intake may even be more advantageous than Se biomarkers, which may be affected by Se own chemical species [56], smoking and other lifestyle variables [66], other nutrients (such as methionine) [67], and drug use [66]. Studying the relationship between dietary Se, serum Se, and T2D could help us to understand this topic more comprehensively. Population bias is another potential limitation of this analysis. Participants undergoing medical health surveys may not be able to confirm the representation of the overall population. However, the relationship between dietary Se and T2D was successfully measured by using large sample size, multivariate logistic regression model, and a comprehensive adjustment of confounding factors. The prevalence of T2D in the targeted population was 20.4%. That is much higher than the computed value of the representative sample of Chinese adults in 2010, concluding a gradual increase in the prevalence of T2D.

## 5. Conclusion

The present study adds to the evidence of a positive correlation between dietary Se intake and incidence of T2D in the target population. The epidemiological evidence for a diabetogenic effect of Se suggests that Se rich foods should be consumed carefully, considering individual dietary requirements. Further work aimed to investigate the potential links between dietary Se and exposure of T2D needs to be further assessed on a priority basis.

## Figures and Tables

**Table 1:** Characteristics of participants according to diabetes status (*N*=8,824).

Selected characteristics	No diabetes	Diabetes	*P*
	*N*=7,020	*N*=1,804	
Age at recruitment (years)	49.74 ± 10.17	54.90 ± 9.32	<0.001
BMI (kg/m^2^)	24.67 ± 3.44	26.14 ± 3.60	<0.001
WC (cm)	84.80 ± 10.13	89.88 ± 9.85	<0.001
Education (%)			<0.001
No formal education	1.4	2.9	
Elementary school	4.4	7.0	
Middle school	21.8	28.4	
High school/secondary technical school	32.6	31.0	
Technical school/college	33.0	24.2	
Postgraduate degree or above	0.8	0.3	
Male (%)	34.0	44.3	<0.001
Female (%)	66.0	55.7	<0.001
Exercised regularly (%)	45.3	53.5	<0.001
Current smokers (%)	54.7	46.5	<0.001
Current drinker (%)	35.0	32.9	<0.001
Family history of diabetes (%)	13.2	24.2	<0.001
Hypertension (%)	76.634.1	59.1	<0.001
Coronary heart disease (%)	15.9	29.2	<0.001
Hyperlipidemia (%)	20.2	38.9	<0.001
Dietary Se intake (*μ*g/day)	51.81 ± 22.97	54.87 ± 26.35	0.001
HOMA-IR	2.0 ± 2.2	4.14 ± 4.87	<0.001
HOMA-*β*	89.85 ± 96.29	61.04 ± 65.34	<0.001
Body fat	30.28 ± 5.69	31.29 ± 6.10	<0.001
Energy	2239.25 ± 655.93	2216.02 ± 688.26	0.184

BMI: body mass index; WC: waist circumference; Se: selenium; HOMA-IR: homeostasis model assessment of insulin resistance; HOMA-*β*: homeostasis model assessment beta. Data are mean ± standard deviation, unless otherwise indicated. *P* values are for test of difference between different diabetic statuses.

**Table 2:** Food sources of selenium consumed by North Chinese people.

Food	%
Rice	13.38
Wheat	22.36
Potatoes	2.09
Beans	0.71
Vegetables	5.19
Fruits	1.51
Livestock and poultry	20.99
Milk	2.20
Eggs	11.30
Fish	18.86
Snacks	0.70
Beverages	0.35
Ice cream	0.36

**Table 3:** Characteristics of participants according to energy-adjusted quintiles of dietary selenium intake (*N*=8,824).

Selected characteristics	Quintiles of dietary Se intake (*μ*g/day)					*P*_trend_
	Q1	Q2	Q3	Q4	Q5	
	*N*=1,764	*N*=1,766	*N*=1,765	*N*=1,764	*N*=1,765	
Age at recruitment (years)	50.3	50.99	50.69	51.14	50.88	0.126
BMI (kg/m^2^)	25.25	24.86	24.83	24.87	25.03	0.002
WC (cm)	86.84	85.51	85.03	85.49	86.31	<0.001
Education (%)						<0.001
No formal education	2.0	1.5	1.5	1.6	1.9	
Elementary school	7.4	5.0	4.8	4.0	3.2	
Middle school	25.7	22.8	23.3	21.7	22.3	
High school/secondary technical school	30.9	33.6	31.4	32.9	32.6	
Technical school/college	28.2	29.9	32.0	33.0	33.2	
Male (%)	40.5	31.3	30.3	34.9	43.6	<0.001
Female (%)	59.5	68.7	69.7	65.1	56.4	<0.001
Exercised regularly (%)	44.2	47.0	47.1	49.7	46.8	0.032
Current smokers (%)	20.5	15.3	14.8	15.9	21.2	<0.001
Current drinker (%)	35.0	32.0	32.8	33.0	40.1	0.046
Family history of diabetes (%)	14.8	16.5	15.6	15.8	14.7	0.575
Hypertension (%)	41.6	37.6	38.4	37.9	40.4	0.065
Coronary heart disease (%)	19.3	17.8	20.1	18.2	17.8	0.288
Hyperlipemia (%)	25.1	24.3	24.3	23.5	22.7	0.528
HOMA-IR	2.46	2.26	2.61	2.38	2.60	0.051
HOMA-*β*	90.59	83.70	84.19	82.74	79.96	0.132
Body fat	30.40	30.77	30.67	30.59	29.99	<0.001
Energy	2633.57	2170.74	2026	1987.91	2354.39	0.184

BMI: body mass index; WC: waist circumference; Se: selenium; HOMA-IR: homeostasis model assessment of insulin resistance; HOMA-*β*: homeostasis model assessment beta. Data are mean ± standard deviation, unless otherwise indicated. *P* values are for test of difference across all quartiles of selenium intake.

**Table 4:** Odds ratios (95% CI) of incident type 2 diabetes by quintiles of dietary selenium intake in the total population, gender, and BMI subgroups.

Selected characteristics	Quintiles of dietary Se intake (*μ*g/day)					*P*_trend_
	Q1	Q2	Q3	Q4	Q5	
Range of Se intake	≤39.24	39.25–46.45	46.46–52.57	52.58–60.68	>60.69	
No. of cases	285	369	338	378	434	
Total population						
Reduced model OR	1.00	1.48 (1.23–1.76)	1.35 (1.13–1.62)	1.51 (1.26–1.80)	1.74 (1.46–2.07)	<0.001
^*∗*^Multivariable adjusted OR	1.00	1.34 (1.11–1.63)	1.20 (0.99–1.47)	1.39 (1.14–1.69)	1.66 (1.38–1.99)	<0.001
Sex subgroup						
Male	1.00	1.62 (1.23–2.14)	1.21 (0.91–1.62)	1.29 (0.98–1.71)	1.45 (1.12–1.89)	0.024
Female	1.00	1.33 (1.03–1.72)	1.39 (1.08–1.80)	1.73 (1.34–2.23)	2.14 (1.65–2.77)	<0.001
BMI subgroup						
BMI < 25	1.00	1.57 (1.16–2.13)	1.42 (1.04–1.93)	1.75 (1.30–2.36)	2.12 (1.57–2.85)	<0.001
BMI ≥ 25	1.00	1.32 (1.06–1.62)	1.19 (0.94–1.55)	1.38 (1.10–1.71)	1.57 (1.24–1.98)	<0.001

Se: selenium; OR: odds ratio. Reduced model: age, sex, BMI, waist. ^*∗*^Multivariable adjusted: age, sex, BMI, WC, education smoking (past, current, never), alcohol intake, energy intake (nonalcohol), body fat, education, family history of diabetes, exercise, hypertension, and coronary heart disease.

**Table 5:** Insulin resistance according to dietary Se intake.

	Q1	Q2	Q3	Q4	Q5
Number	*N*=1764	*N*=1766	*N*=1765	*N*=1764	*N*=1765
Se (*μ*g/day)	≤ 39.24	39.25–46.45	46.46–52.57	52.58–60.68	>60.69
	*r* (*p*)	*r* (*p*)	*r* (*p*)	*r* (*p*)	*r* (*p*)
HOMA-IR	−0.009885 (0.755)	−0.021618 (0.496)	0.012121 (0.698)	0.030804 (0.330)	−0.010850 (0.743)
HOMA-*β*	0.022180 (0.442)	0.030418 (0.339)	−0.006698 (0.830)	0.013095 (0.678)	0.016879 (0.611)

Partial correlations between energy-adjusted dietary Se (*μ*g/day) quintiles and insulin resistance after controlling for age and BMI. HOMA-IR: homeostasis model assessment of insulin resistance; HOMA-*β*: homeostasis model assessment of *β* cell function, *r*: partial correlation coefficient.

**Table 6:** Correlations between dietary Se intake and glucose metabolism.

	*r*0 (*p*)	*r*1 (*p*)
FINS (pmol/L)	−0.000105 (0.994)	0.057944 (0.978)
FBG (mmol/L)	0.046490 (0.001)	0.053343 (0.001)
HbA1c	0.057944 (0.001)	0.053372 (0.001)

Partial correlations between dietary Se intake (*μ*g/day) and insulin resistance after controlling for age, sex, BMI. FINS: fasting insulin; FBG: fasting blood glucose; HbA1c: haemoglobin A1c; *r*0: correlation coefficient; *r*1: partial correlation coefficient.

**Table 7:** Causal associations between dietary Se and T2D in HDNNCDS.

Potential mediator	The total effects (95% CI)	Proportion via mediation (95% CI)	Prop. mediated
			*P* value
FBG	0.00086 (0.00046, 0.00127)	0.54980 (0.29877, 0.86451)	<0.001
HbA1c	0.00063 (0.00016, 0.00116)	0.79426 (0.41340, 2.41389)	0.006
FINS	0.00077 (0.00027, 0.00132)	−0.00020 (−0.07005, 0.06494)	0.986
HOMA-IR	0.00076 (0.00024, 0.00119)	0.04835 (−0.08660, 0.21473)	0.328
HOMA-*β*	0.00077 (0.00029, 0.00124)	0.04563 (−0.03614, 0.17352)	0.202

FBG: fasting blood glucose; HbA1c: haemoglobin A1c; FINS: fasting insulin; HOMA-IR: homeostasis model assessment of insulin resistance; HOMA-*β*: homeostasis model assessment of *β* cell function.

## Data Availability

All research data are available and will be provided on request to the corresponding author.
